# Optimizing linkage and retention to hypertension care in rural Kenya (LARK hypertension study): study protocol for a randomized controlled trial

**DOI:** 10.1186/1745-6215-15-143

**Published:** 2014-04-27

**Authors:** Rajesh Vedanthan, Jemima H Kamano, Violet Naanyu, Allison K Delong, Martin C Were, Eric A Finkelstein, Diana Menya, Constantine O Akwanalo, Gerald S Bloomfield, Cynthia A Binanay, Eric J Velazquez, Joseph W Hogan, Carol R Horowitz, Thomas S Inui, Sylvester Kimaiyo, Valentin Fuster

**Affiliations:** 1 Icahn School of Medicine at Mount Sinai, One Gustave L. Levy Place, Box 1030, 10029 New York, USA; 2 Department of Medicine, Moi Teaching and Referral Hospital, Nandi Road, P.O. Box 3-30100, Eldoret, Kenya; 3 Department of Behavioural Sciences, School of Medicine, Moi University College of Health Sciences; 4 Department of Biostatistics and Center for Statistical Sciences, Brown University, 121 South Main Street, 02903 Providence, Rhode Island, USA; 5 Department of Medicine and Regenstrief Institute, Inc, Indiana University School of Medicine, 410 W. 10th Street, 46202 Indianapolis, Indiana, USA; 6 Global Health Institute, Duke University, 310 Trent Drive Durham North Carolina 27710, USA; 7 Department of Epidemiology & Nutrition, School of Public Health, Moi University, P.O. Box 3-30100 Eldoret, Kenya; 8 Department of Medicine, Moi University College of Health Sciences, P.O. Box 3-30100 Eldoret, Kenya; 9 Clinical Research Institute, Duke University, 2400 Pratt Street, DUMC 3850, 27705 Durham, North Carolina, USA; 10 Centro Nacional de Investigaciones Cardiovasculares, 28029 Madrid, Spain

**Keywords:** Hypertension, Linkage to care, Retention in care, Community health workers, Tailored behavioral communication, Smartphone technology, Cost-effectiveness

## Abstract

**Background:**

Hypertension is the leading global risk factor for mortality. Hypertension treatment and control rates are low worldwide, and delays in seeking care are associated with increased mortality. Thus, a critical component of hypertension management is to optimize linkage and retention to care.

**Methods/Design:**

This study investigates whether community health workers, equipped with a tailored behavioral communication strategy and smartphone technology, can increase linkage and retention of hypertensive individuals to a hypertension care program and significantly reduce blood pressure among them. The study will be conducted in the Kosirai and Turbo Divisions of western Kenya. An initial phase of qualitative inquiry will assess facilitators and barriers of linkage and retention to care using a modified Health Belief Model as a conceptual framework. Subsequently, we will conduct a cluster randomized controlled trial with three arms: 1) usual care (community health workers with the standard level of hypertension care training); 2) community health workers with an additional tailored behavioral communication strategy; and 3) community health workers with a tailored behavioral communication strategy who are also equipped with smartphone technology. The co-primary outcome measures are: 1) linkage to hypertension care, and 2) one-year change in systolic blood pressure among hypertensive individuals. Cost-effectiveness analysis will be conducted in terms of costs per unit decrease in blood pressure and costs per disability-adjusted life year gained.

**Discussion:**

This study will provide evidence regarding the effectiveness and cost-effectiveness of strategies to optimize linkage and retention to hypertension care that can be applicable to non-communicable disease management in low- and middle-income countries.

**Trial registration:**

This trial is registered with (NCT01844596) on 30 April 2013.

## Background

Cardiovascular disease (CVD) is the leading cause of mortality in the world, with 80% of CVD deaths occurring in low- and middle-income countries (LMICs) [1]. Hypertension, a major risk factor for ischemic heart disease, heart failure, and stroke [2], is the leading global risk for mortality [3]. The global cost of suboptimal blood pressure (BP) is estimated to reach nearly $1 trillion over the next decade [4]. Unless adequately controlled, hypertension will continue to be responsible for significant morbidity and mortality worldwide [5].

### Rationale for a focus on linkage and retention to care

Hypertension awareness, treatment, and control rates are low worldwide [6]. In Kenya, hypertension treatment and control rates have been reported at below 15% and 5%, respectively [7-9]. Given that hypertension may be asymptomatic, linkage and retention to care and medication adherence are particularly difficult challenges [10]. Delays in seeking hypertension care have been shown to be associated with increased mortality [11]. Thus, early linkage to hypertension care and successful retention to clinical services are critical components of hypertension management.

Studies of hypertension programs in sub-Saharan Africa have suggested that addressing financial barriers, provider-patient communication, and education may improve linkage, retention, and medication adherence [12-14]. Although preliminary qualitative research in western Kenya has revealed that community members understand that hypertension can cause significant morbidity and mortality [15], there are few known specific strategies to optimize linkage and retention to hypertension care in this setting.

### Components of the proposed intervention to optimize linkage and retention to care

Community health workers (CHWs) are members of a community who have received basic training to supply community members with access to health and social services, to educate individuals about various health issues, and to support overall community development [16]. CHWs have been utilized in communicable disease and maternal/child health programs [17]. However, they are only recently being deployed and evaluated in the context of non-communicable diseases [18], and the impact of CHWs on linkage and retention to hypertension care in Africa is relatively unknown.

Motivational interviewing and tailored communication-strategies intended to tailor interventions based on individual-specific behavioral assessments-have been shown to improve a variety of health behaviors [19,20]. In addition, mobile technology-based tools have the potential to improve the scope and efficiency of CHWs, and have demonstrated benefit for communicable diseases [21,22]. However, these strategies have not been rigorously evaluated in the context of hypertension management in LMICs. Thus, we plan to utilize a multidisciplinary implementation research approach [23] to develop and evaluate innovative community-based strategies, supported by mobile technology, to optimize linkage and retention to a hypertension management program in western Kenya.

### Aims

The central hypothesis of this study is that CHWs equipped with a tailored behavioral communication strategy, with or without smartphone technology, can increase linkage and retention of hypertensive individuals to a hypertension care program and thereby significantly reduce BP among these patients, when compared to usual care. We further hypothesize that these interventions will be cost-effective. Thus, the aims of this study are threefold. First, we aim to identify the facilitators and barriers to linking and retaining individuals with high BP to a hypertension care delivery program, using qualitative research methods. With this information, we will develop a communication strategy and a smartphone-based tool linked to an electronic health record. Second, we aim to evaluate the incremental effectiveness of the communication strategy and the smartphone-based tool in improving linkage and reducing BP among hypertensive patients. This will be done by conducting a three-arm cluster randomized controlled trial comparing: 1) usual care (CHWs with standard training on recruitment of individuals with any chronic condition); 2) CHWs with a communication strategy; and 3) CHWs with the communication strategy and equipped with smartphone technology. The co-primary outcome measures will be: 1) documented linkage to care, and 2) one year change in systolic BP (SBP) among those with hypertension. Third, we aim to evaluate the incremental cost-effectiveness of each intervention arm of the cluster randomized controlled trial, in terms of costs per unit decrease in BP and costs per disability-adjusted life year (DALY) gained.

## Methods/Design

### Setting

**Figure 1:**
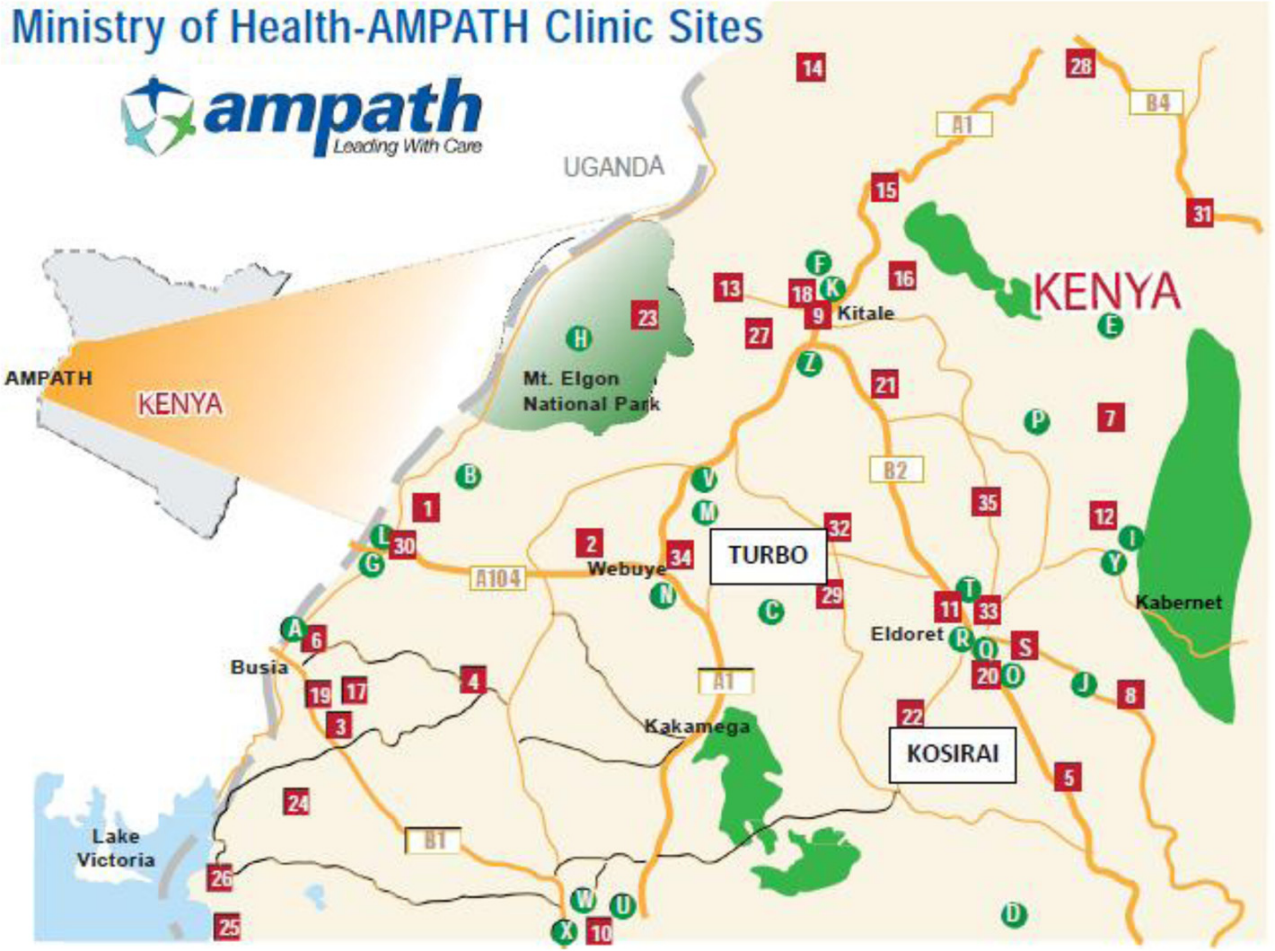
AMPATH centers in Kenya numbered 1 to 35 and lettered A to Z; Kosirai and Turbo Divisions highlighted.

The United States Agency for International Development-Academic Model Providing Access to Healthcare Partnership (AMPATH) was initiated in Kenya in 2001 and has established a HIV care system in western Kenya that serves over 100,000 patients [24]. Based on that foundation, and in partnership with the Government of Kenya, AMPATH is expanding its clinical scope of work to include hypertension [25]. This study will be conducted within the AMPATH infrastructure in western Kenya, in the Kosirai and Turbo Divisions (Figure 1). Each Division is geographically and administratively divided into Community Units of approximately 5,000 individuals, with 9 units in Kosirai and 15 units in Turbo. Each Division has one rural health center staffed primarily by non-physician clinical officers trained to deliver a range of clinical health services [26], decentralized rural dispensaries staffed by nurses, and CHWs who are assigned to specific units. There has been a longstanding and positive relationship among AMPATH, the healthcare providers, and these communities [24,27,28]. The protocol has been approved by the institutional review boards of all participating institutions (Additional file 1), and the study is a registered on http://www.clinicaltrials.gov (identifier NCT01844596). Informed consent will be obtained from all study participants using a written informed consent form. During the consent process, participants will have the opportunity to request information and pose questions or concerns about their participation in the study.

### Conceptual framework

**Figure 2:**
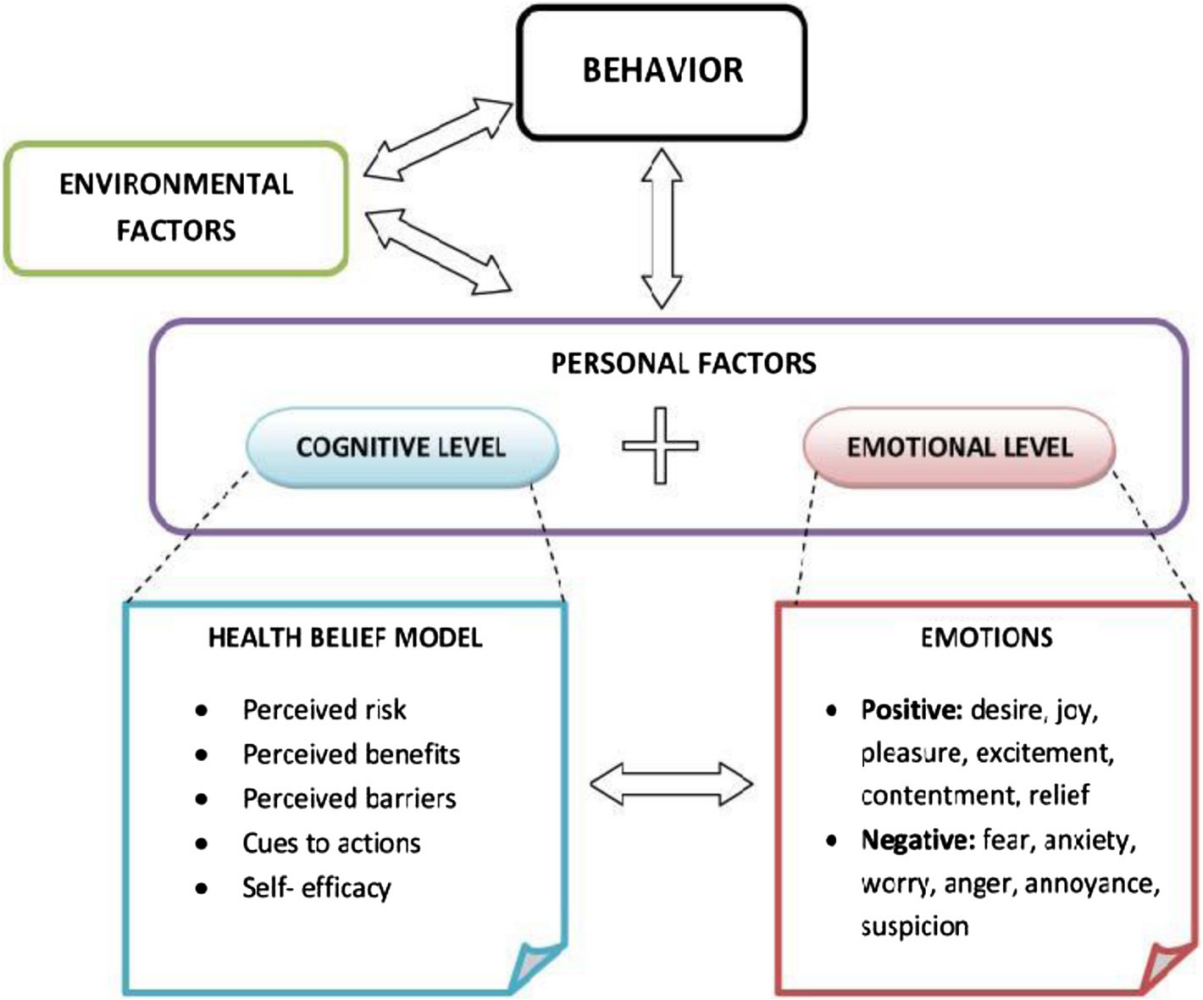
Modified Health Belief Model: personal (cognitive and emotional) and environmental factors.

Many behavior change models have been developed that focus on various types of health-related behavior and disease entities. In this study, we use the Health Belief Model, modified by incorporating the additional impact of emotional and environmental factors on behavior (Figure 2) [29]. The Health Belief Model is primarily a cognitive model based on the domains of perceived risk, perceived benefits, perceived barriers, cues to action, and self-efficacy [30]. While the Health Belief Model has been successfully applied to hypertension-related research in a variety of settings and populations [31,32], we have expanded the model to incorporate the complex interactions among cognition, emotion, environment, and behavior. Emotional factors include desires, aspirations, fears, and worries that may directly motivate action or serve as a powerful lens to weigh advantages and disadvantages of alternative actions [33]. Environmental factors include socioeconomic factors, costs, political constraints, and cultural norms, which may facilitate or constrain an individual’s behavior.

### Facilitators and barriers to linkage and retention to hypertension care

We will use a combination of qualitative research methods, including traditional community assemblies (*mabaraza*) and focus group discussions (FGDs), to identify the facilitators and barriers to linkage and retention to hypertension care. In East Africa, the *mabaraza* are used to address a wide variety of situations, ranging from local disputes to exchange of information. This unique and novel qualitative research setting has been used as a form of participatory action research related to HIV care [34]. The *mabaraza* allow us to organize large and heterogeneous groups of individuals, which complement the purposive sampling inherent in FGDs.

For all qualitative sessions, we have developed moderator guides, which have been used by trained moderators fluent in the local languages. We have thus far conducted 6 *mabaraza* and 17 FGDs using purposive sampling by age, sex, occupation, and distance from nearest health facility. Participatory techniques have been used to elicit emotional elements and promote group interactions [35]. All sessions have been audio-recorded, transcribed, and translated into English. Content analysis of the transcripts will be performed using both deductive (*a priori*) and inductive (emerging) codes [36]. The coded items will be grouped together into distinct themes, and relationships among these themes will be formulated.

Using the data gathered in the formative qualitative sessions, we will use a participatory, iterative design process to develop a tailored behavioral communication strategy for CHWs to use as they interact with patients and their families [37]. CHWs will be trained to engage in practical, motivating, proactive problem-solving with patients around such issues as transportation, clinic scheduling, home responsibilities, stigma concerns, and other issues that may be uncovered by the qualitative research. We will develop both a hard copy version and a smartphone version to be used by CHWs.

The smartphone version will be linked to the electronic health record and will have two functionalities: 1) ability to provide each CHW with an automatically updated list of individuals requiring follow up; and 2) the ability to provide real-time decision support based on the communication strategy, using data collected by the CHW during a patient encounter. The decision support will use branching logic and decision trees based on specific motivational messages, as well as simple clinical care algorithms appropriate for CHWs. The smartphone technology will allow for alternative messaging modalities, such as images and recordings (both audio and visual).

### Cluster randomized controlled trial

We will conduct a cluster randomized controlled trial with three intervention arms: 1) usual care - CHWs with standard training on recruitment of individuals with any chronic condition (‘UC’); 2) CHWs with a tailored behavioral communication strategy (‘TBCS’); and 3) CHWs with a communication strategy who are also equipped with smartphone technology (‘TBCS-ST’). The unit of randomization will be the community unit, since randomization by CHW or patient would be at risk for contamination, as all CHWs and nearly all patients within one community unit are affiliated with the same dispensary. Randomization will be stratified by division, so that the 9 units of Kosirai will be randomized separately from the 15 units of Turbo. The units will be randomly allocated to one of the three intervention arms. The randomization process will be conducted centrally by biostatisticians at Brown University (Providence, United States). We will compare key variables (such as age, sex, severity of initial SBP, different treatment regimens, previous history of hypertension treatment, socioeconomic status, body mass index, physical activity, alcohol consumption, and tobacco use) to ensure balance among the randomized groups, and make adjustments in our treatment effect estimates as needed using logistic regression adjustment.

### Study participants

**Figure 3:**
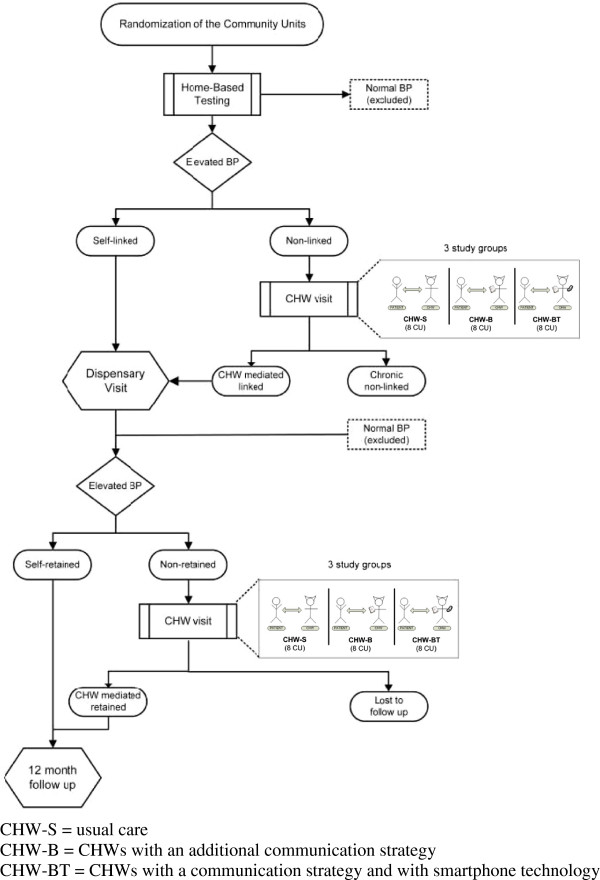
**Classification of participants in trial.** BP = Blood Pressure; CHW-S = usual care arm; CHW-B = tailored behavioral communication strategy arm; CHW-BT = communication strategy and smartphone technology arm; CU- Community Unit.

Home-based BP testing using automatic BP machines has been initiated by AMPATH in both the Kosirai and Turbo divisions, with a plan to cover one-third of each community unit’s adult population every year. Inclusion criteria for this study will be all adult individuals with elevated BP (SBP >140 or diastolic BP (DBP) >90) during home-based testing, who will be assigned a unique medical record number and referred to the local dispensary for further evaluation (Figure 3). Exclusion criteria will be individuals without elevated BP, those who are acutely ill and require immediate medical attention at the time of testing, and individuals who do not provide informed consent. Enrollment will occur for one year in each unit.

An individual who does not present to the dispensary within one month will be defined as ‘non-linked’ and will be identified by the dispensary nurse. At that point in time, a CHW will be assigned to visit that individual to encourage linkage to care. Those who do not link to care within one month of the second CHW visit will be considered ‘chronic non-linked’. Individuals who ultimately link to care will be characterized as ‘self-linked’ (linked on their own without CHW intervention) or ‘CHW-mediated linked’. At the dispensary, each individual who has linked will have a repeat BP measured, and those with repeat elevated BP will be entered into the hypertension management program as patients. Patients will be managed according to the AMPATH hypertension protocol that is derived from consensus guidelines for hypertension management, using drugs contained in the Kenyan national formulary [38-40]. Patients who miss a clinic appointment for more than one month will be considered ‘non-retained’. Patients who are retained in care will be defined as either ‘self-retained’ (retained on their own without CHW intervention) or ‘CHW-mediated retained’. An individual will be considered ‘lost to follow up’ if they does not return to the clinic for three months despite CHW visits. The 12-month follow up BP will be measured in the dispensary, in order to mimic real-world practice.

### Intervention and control

In the UC arm, the CHW will first measure the individual’s BP. If it is elevated (SBP >140 or DBP >90), the CHW will refer the individual to the dispensary for further evaluation and management, as per the usual care protocol.

In the TBCS arm, the CHW will measure the individual’s BP and then will engage in behavioral, clinical, and environmental assessments. Based on the behavioral assessment, the CHW will employ the communication strategy consisting of tailored behavioral and motivational messages, as described above. Depending on the severity of the clinical assessment, the CHW will either refer or accompany high-risk patients to the dispensary. The environmental assessment will evaluate socioeconomic barriers to care-seeking, and the CHW will provide this information to the nurses in the dispensary.

In the TBCS-ST arm, the CHW will conduct all assessments described above; however, s/he will also be equipped with a smartphone that has real-time decision support and data entry that is linked to the electronic health record. Thus, the smartphone will provide the tailored messaging and specific recommendations based on inputs from the assessments. The smartphone technology would allow for alternative messaging modalities, such as images and recordings (audio and visual), to ensure its applicability to a population speaking diverse languages and with different literacy levels. If a patient does successfully go to the dispensary for the linkage visit, this visit will be entered into the electronic health record and we will program the system to immediately send a positive reinforcement message to the CHW.

### Outcomes

The co-primary outcome measures will be: 1) documented linkage to care, defined as a confirmed dispensary visit within one month of either home-based testing (self-linked) or a CHW visit (CHW-mediated linked), and 2) one-year change in SBP among those with hypertension. Our ultimate goal is to implement an intention-to-treat comparison of change in SBP among confirmed hypertensive individuals. However, confirmation of hypertensive status requires at least two BP measurements, and this requires successful linkage to care (Figure 3). Hence, we have opted for the co-primary outcome approach. We will compare the proportion linked to care between the three study arms using conditional logistic regression. For the SBP outcome, we will implement an intention-to-treat comparison of change in SBP between the three study arms. For those who are not successfully linked to care, we will use imputed SBP values. Full details of our approach to imputation appear in Additional file 2.

### Statistical power

The study is powered on the linkage-to-care outcome in order to preserve the properties of randomization at the community unit level and avoid bias due to differential selection into care by the linkage intervention. In light of AMPATH’s pilot experience with home-based testing and linkage to hypertension care [10], we anticipate that the usual care arm will have 40% linkage (self-linked plus CHW-mediated linked). The study is powered to detect an absolute difference of 20% in percent linkage to care between each of the three treatment arms (expected 60% linkage in the TBCS arm and 80% linkage in the TBCS-ST arm). It is expected that 226 people will be referred to the dispensary per community unit per year, based on the following: 1) each community unit has a population of approximately 5,000; 2) 45.3% of the population is above the age of 20 [41]; 3) one-third of the population will be screened during the year; and 4) approximately 30% of adults will have an elevated SBP at the home-based testing [10].

**Figure 4:**
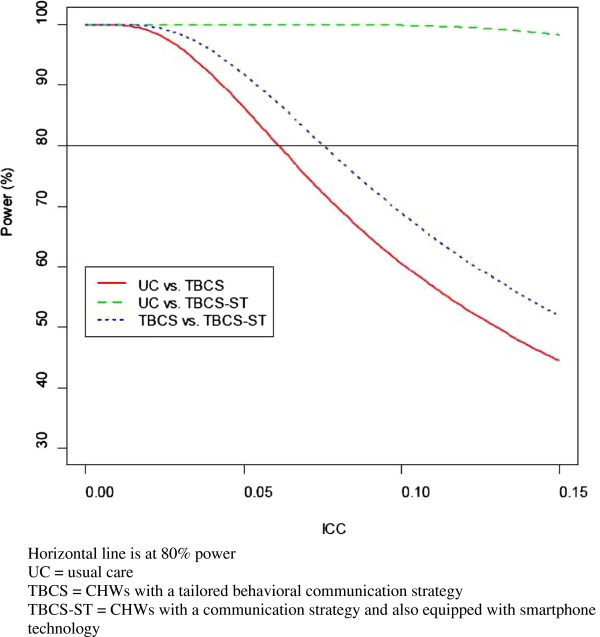
**Power to detect differences in the proportion of patients linked to care over a realistic range of ICC coefficients.** Horizontal line is at 80% power. CHWs, community health workers; ICC, intraclass correlation; TBCS, CHWs with a tailored behavioral communication strategy; TBCS-ST, CHWs with a communication strategy and also equipped with smartphone technology; UC, usual care.

Our power calculations set overall Type I error rate at 5% (alpha = 0.05), and use a Bonferroni correction to permit all pairwise comparisons between treatment arms. To account for cluster effects, we consider scenarios where intraclass correlation (ICC) ranges from 0 to 0.15 [42]. Plots demonstrating power for each of the pairwise treatment arm comparisons across different values of ICC reveal that our pairwise comparisons have over 80% power over a large range of ICC values (Figure 4).

### Cost-effectiveness analysis

For each intervention arm, costs from the societal perspective will be captured using validated cost questionnaires and will include all relevant labor, materials, supplies, and contracted services costs for all activities required to deliver the interventions [43,44]. Only incremental (variable and fixed) costs will be included in the analysis. We will also identify which activities drive the overall costs, and how costs would change if specific activities are added or eliminated. We will also identify potential cost offsets (reductions in health services utilization as a result of participation in a specific arm) using health service utilization data contained within the electronic health record and based on utilization and cost questionnaires that participants will take at baseline and 12 months. We will thus quantify the net costs of participation in each intervention arm.

Once costs and effectiveness are calculated for each intervention arm, we will then generate incremental cost-effectiveness ratios (ICERs) following the approach described in the literature [43]. First, costs and effectiveness measures will be tabulated for each strategy in order of increasing costs. After removing dominated interventions, we will present the final ICERs and provide comparisons to other CVD interventions and to other interventions targeting this population after first converting the results to international dollars using World Health Organization conversion rates [45]. Our results will also be presented in the form of cost-effectiveness acceptability curves [46], which will show the probability that each strategy is cost-effective for a range of monetary values that a decision-maker might be willing to pay for a unit change in effectiveness. In addition, we will perform one-way (and n-way) sensitivity analyses, in which we will examine the effect of changing one (or n) of the model parameters, holding all other parameters constant.

We will evaluate the incremental cost-effectiveness of each intervention arm, both in terms of costs per unit decrease in BP and in terms of costs per unit reduction in CVD risk by extrapolating one-year BP reductions to CVD risk reductions based on the QRISK™2-2011 CVD risk calculator (University of Nottingham, Nottingham, United Kingdom) specific for Black African populations [47]. We will then present costs per DALY saved by extrapolating these reductions to all-cause mortality, using an approach we have previously developed, with care to note all assumptions required [48].

## Discussion

The global burden of hypertension and other non-communicable diseases is substantial and increasing, especially in LMICs. However, insufficient data exist regarding effective health care delivery practices in these settings. Each step in the implementation pathway, including linkage and retention to care, can benefit from evidence-based approaches. The LARK hypertension study has been designed with these objectives in mind, and offers several unique and innovative elements. First, we are expanding the traditional Health Belief Model to include emotional and environmental factors that influence health behavior. Second, we are utilizing the *mabaraza* form of qualitative inquiry and actively pursuing a participatory methodology throughout all phases of the research. Third, we are evaluating the use of mobile technology in a novel domain of non-communicable disease care delivery. Fourth, we are embedding the research within a foundational partnership of academic institutions, communities, and local government, to ensure that the program meets the needs of all stakeholder groups. Finally, we are working within the existing structure of primary care delivery that is established by the government.

We aim to demonstrate how the infrastructure and strategies that have been established for the control of communicable diseases-including community-based screening, task redistribution within teams, partnerships with local providers, and medical informatics- can serve as a foundation for an integrated delivery system approach to the control of non-communicable chronic conditions [49]. Thus, the results of this project can serve as a platform to be used for other non-communicable diseases, such as stroke, diabetes, respiratory disease, cancer, and mental illness. While this research is situated within a particular sociocultural and institutional context, we aim to produce generalizable methods and results that can be applied in other settings. We therefore hope that this project will provide a model for the evaluation of other new approaches to non-communicable disease management in LMICs.

## Trial status

The cluster randomized controlled trial portion of this study has not yet begun. The qualitative portion of the study is currently ongoing.

## Abbreviations

AMPATH: academic model providing access to healthcare partnership; BP: blood pressure; CHW: community health worker; CVD: cardiovascular disease; DALY: disability-adjusted life year; DBP: diastolic blood pressure; FGD: focus group discussion; ICC: intraclass correlation; ICER: incremental cost-effectiveness ratio; LMICs: low- and middle- income countries; SBP: systolic blood pressure; TBCS: tailored behavioral communication strategy; TBCS-ST: tailored behavioral communication strategy with smartphone technology; UC: usual care.

## Competing interests

The authors declare that they have no competing interests.

## Authors’ contributions

VF is the principal investigator of the study, was responsible for and conceived the concept and design of the overall study, had ultimate oversight over the study design and conduct of the study, and reviewed the manuscript critically for content. RV contributed to the content and design of the overall study and drafted the manuscript. JHK contributed to the content and design of the overall study and reviewed the manuscript critically for content. VN led the design of the qualitative portion of the study and reviewed the manuscript critically for content. AKD contributed to the randomization protocol and statistical analysis plan and reviewed the manuscript critically for content. MCW led the design of the information technology component of the study and reviewed the manuscript critically for content. EAF designed the cost-effectiveness analysis and reviewed the manuscript critically for content. DM contributed to the design of the community health worker intervention and reviewed the manuscript critically for content. COA contributed to the overall protocol development and reviewed the manuscript critically for content. GSB contributed to the content and design of the study and reviewed the manuscript critically for content. CAB contributed to the overall protocol development and reviewed the manuscript critically for content. EJV contributed to the content and design of the study and reviewed the manuscript critically for content. JWH led the design of the randomization protocol and statistical analysis plan and reviewed the manuscript critically for content. CRH contributed to the content and design of the study and reviewed the manuscript critically for content. TSI contributed to the content and design of the study and reviewed the manuscript critically for content. SK contributed to the content and design of the study and reviewed the manuscript critically for content. All authors read and approved the final manuscript.

## Supplementary Material

Additional file 1 Is the list of all IRBs that have approved the protocol. Click here for file

Additional file 2: Figure S1 Schematic illustrating the strategy for estimating intention-to-treat effect on change in SBP among those diagnosed with hypertension. Light gray boxes represent individuals with suspected hypertension at time 0; white boxes represent patients with hypertension; dark gray boxes represent those without hypertension. Abbreviations as in the Appendix; FU = follow-up. Click here for file
