# Supplementary material for: Optimizing linkage and retention to hypertension care in rural Kenya (LARK hypertension study): study protocol for a randomized controlled trial
Source: Trials. 2014 Apr 27;15:143. doi: 10.1186/1745-6215-15-143 (PMC4113229; doi:10.1186/1745-6215-15-143)
Supplement: Additional file 1 — Is the list of all IRBs that have approved the protocol. [file 1745-6215-15-143-S1.pdf]

List of approvals from ethical bodies at all institutions

**Institution**	**Status**	**Justification**
**Icahn School of Medicine at Mount Sinai** – Institutional Review Board (IRB)	Approved	Most recent annual approval was obtained in April 2013. Renewal application has been submitted and approval is expected.
**Moi Teaching and Referral Hospital and Moi University –** Institutional Research and Ethics Committee (IREC)	Approved	Most recent annual approval was obtained in March 2014.
**Duke University** – Duke University Health System Institutional Review Board (DUHS IRB)	Exempt from IRB approval	DUHS IRB determined that, for Duke investigators, the study protocol meets the definition of research not involving human subjects as described in 45 CFR46.102(f), 21 CFR 56.102€ and 21 CFR 812.3(p) and satisfies the Privacy Rule as described in 45CFR164.514.
**Brown University –** Institutional Review Board (Brown IRB)	Exempt from IRB approval	Brown University has deferred to the Mount Sinai IRB for review and continuing oversight of the human subjects research for this protocol.
**Indiana University** – Institutional Review Board (IU IRB)	Exempt from IRB approval	IU has deferred to the Mount Sinai IRB for review and continuing oversight of the human subjects research for this protocol.
