# Supplementary material for: Optimizing linkage and retention to hypertension care in rural Kenya (LARK hypertension study): study protocol for a randomized controlled trial
Source: Trials. 2014 Apr 27;15:143. doi: 10.1186/1745-6215-15-143 (PMC4113229; doi:10.1186/1745-6215-15-143)
Supplement: Additional file 2: Figure S1 — Schematic illustrating the strategy for estimating intention-to-treat effect on change in SBP among those diagnosed with hypertension. Light gray boxes represent individuals with suspected hypertension at time 0; white boxes represent patients with hypertension; dark gray boxes represent those without hypertension. Abbreviations as in the Appendix; FU = follow-up. [file 1745-6215-15-143-S2.zip › 3730023951115190_add2/3730023951115190_add2a.pdf]

Appendix

Supplementary Figure 1 illustrates our approach to this imputation method. At home-based testing (time = 0), individuals who are at risk for hypertension will be identified (SBP_0>140 or DBP_0>90). Of those, a subset will have their BP measured at the dispensary; we denote this by BP_1. We consider those individuals to be linked to care. If SBP_1 > 140 or DBP_1 > 90, a diagnosis of hypertension is made. For those linked to care, SBP_1 and DBP_1 are measured directly. For those not linked to care, we will impute values of SBP_1 and DBP_1 as follows: first, we will use those with observed SBP_1 to fit a regression model of the form $SBP_1 = \alpha + \beta SBP_0 + \theta X + \varepsilon$, where X is a vector of covariates that are predictive of SBP_1 (including DBP_0), and ε is a mean-zero error term; next, we will use the fitted model to generate predicted values of SBP_1* for those who were not measured; third, an analogous model for DBP_1 will be used to generate predicted values for DBP_1*. Finally, we will label those with SBP_1* > 140 or DBP_1* > 90 as hypertensive.

Thus, the number of hypertensives is the number linked to care with measured SBP_1 > 140 or DBP_1 > 90, plus the number not linked to care with imputed SBP_1* > 140 or DBP_1* > 90; we will call this sum $N_M + N_I$ (number measured + number imputed). Among those measured at time = 1, only a subset will complete follow up and have SBP measured at one year (time = 2). In calculating the mean change in SBP at one year, those measured at one year will contribute the observed change $\Delta = SBP_2 - SBP_1$. Everyone else will contribute Δ=0 under the assumption that if hypertension is untreated, SBP will not change (on average). Every patient who does not come to the dispensary within one month of the 12-month clinic visit will be followed up by the appropriate CHW, who will record a 12-month BP at that time.

Heuristically, if we let F=1 for those who complete one-year follow up and F=0 for those who do not, and N_F be the number of participants who have BP measured at 12 months, the intention-to-treat mean can be expressed as $p \times E(\Delta \mid F=1)$, where $p = N_F/(N_M + N_I)$ is the proportion of hypertensives who are measured at the one-year follow up, and $E(\Delta \mid F=1)$ is the mean change in SBP among those who completed the follow up. Hence, the estimated change in SBP within a specific treatment arm is the mean change in SBP among those who are linked to care times the proportion who are actually linked to care. The extreme case helps to illustrate: if no one is linked to care, the mean change is zero. The formal estimation and inference about intention-to-treat means and treatment effects will be computed using an appropriately formulated regression model that allows for cluster effects [1-3]. Uncertainty about imputed SBP at time t=1 will be handled using multiple imputation [4, 5].

<u>References for Appendix</u>

1. Hogan JW, Laird NM: **Intention-to-treat Analyses for Incomplete Repeated Measures Data**. *Biometrics* 1996, **52**:1002-1017.
2. Little R, Yau L: **Intent-to-treat Analysis for Longitudinal Studies With Drop-outs**. *Biometrics* 1996, **52**:1324-1333.
3. Hogan JW, Daniels MJ: **A hierarchical modelling approach to analysing longitudinal data with drop-out and non-compliance, with application to an equivalence trial in paediatric acquired immune deficiency syndrome**. *Applied Statistics* 2002, **51**:1-21.
4. Rubin DB: **Multiple Imputation for Nonresponse in Surveys**. New York: John Wiley and Sons; 1987.
5. Robins J, Wang N: **Inference for imputation estimators**. *Biometrika* 2000, **87**:113-124.

Supplementary Figure 1. Schematic illustrating the strategy for estimating intention-to-treat effect on change in SBP among those diagnosed with hypertension. Light gray boxes represent individuals with suspected hypertension at time 0; white boxes represent patients with hypertension; dark gray boxes represent those without hypertension. Abbreviations as in the Appendix; FU = follow-up.
