# Supplementary figures and images for: Optimizing linkage and retention to hypertension care in rural Kenya (LARK hypertension study): study protocol for a randomized controlled trial
Source: Trials. 2014 Apr 27;15:143. doi: 10.1186/1745-6215-15-143 (PMC4113229; doi:10.1186/1745-6215-15-143)

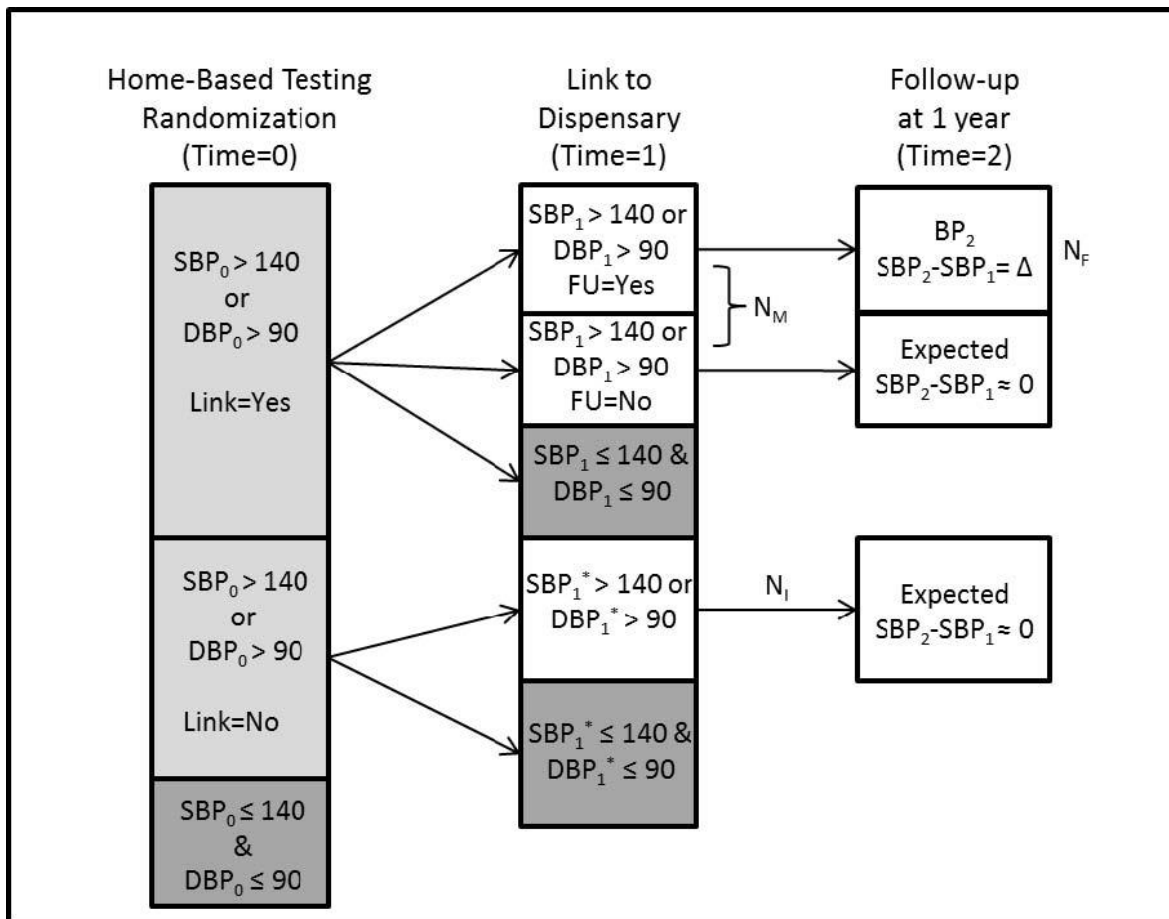

Supplement: Additional file 2: Figure S1 — Schematic illustrating the strategy for estimating intention-to-treat effect on change in SBP among those diagnosed with hypertension. Light gray boxes represent individuals with suspected hypertension at time 0; white boxes represent patients with hypertension; dark gray boxes represent those without hypertension. Abbreviations as in the Appendix; FU = follow-up. [file 1745-6215-15-143-S2.zip › 3730023951115190_add2/3730023951115190_add2b.pdf]
